# A Novel Antidipteran Bacillus thuringiensis Strain: Unusual Cry Toxin Genes in a Highly Dynamic Plasmid Environment

**DOI:** 10.1128/AEM.02294-20

**Published:** 2021-02-12

**Authors:** Nancy Fayad, Zakaria Kambris, Laure El Chamy, Jacques Mahillon, Mireille Kallassy Awad

**Affiliations:** a Laboratory of Food and Environmental Microbiology, Earth and Life Institute, UCLouvain, Louvain-la-Neuve, Belgium; b Laboratory of Biodiversity and Functional Genomics, UR-EGP, Faculty of Science, Université Saint-Joseph de Beyrouth, Beirut, Lebanon; c Biology Department, Faculty of Arts and Sciences, American University of Beirut, Beirut, Lebanon; University of Queensland

**Keywords:** *Bacillus thuringiensis*, Cry toxins, insertion sequences, large plasmids, mosquitocidal, transposable elements

## Abstract

Bacillus thuringiensis has emerged as a major bioinsecticide on the global market. It offers a valuable alternative to chemical products classically utilized to control pest insects. Despite the efficiency of several strains and products available on the market, the scientific community is always on the lookout for novel toxins that can replace or supplement the existing products. In this study, H3, a novel B. thuringiensis strain showing mosquitocidal activity, was isolated from Lebanese soil and characterized *in vivo* at genomic and proteomic levels. H3 parasporal crystal is toxic on its own but displays an unusual killing profile with a higher 50% lethal concentration (LC_50_) than the reference B. thuringiensis serovar israelensis crystal proteins. In addition, H3 has a different toxicity order: it is more toxic to Aedes albopictus and Anopheles gambiae than to Culex pipiens. Whole-genome sequencing and crystal analysis revealed that H3 can produce 11 novel Cry proteins, 8 of which are assembled in genes with an *orf1-gap-orf2* organization, where *orf2* is a potential Cry4-type crystallization domain. Moreover, pH3-180, the toxin-carrying plasmid, holds a wide repertoire of mobile genetic elements that amount to ca. 22% of its size., including novel insertion sequences and class II transposable elements. Two other large plasmids present in H3 carry genetic determinants for the production of many interesting molecules—such as chitinase, cellulase, and bacitracin—that may add up to H3 bioactive properties. This paper therefore reports a novel mosquitocidal Bacillus thuringiensis strain with unusual Cry toxin genes in a rich mobile DNA environment.

**IMPORTANCE** Bacillus thuringiensis, a soil entomopathogenic bacterium, is at the base of many sustainable eco-friendly bioinsecticides. Hence stems the need to continually characterize insecticidal toxins. H3 is an antidipteran B. thuringiensis strain, isolated from Lebanese soil, whose parasporal crystal contains 11 novel Cry toxins and no Cyt toxins. In addition to its individual activity, H3 showed potential as a coformulant with classic commercialized B. thuringiensis products, to delay the emergence of resistance and to shorten the time required for killing. On a genomic level, H3 holds three large plasmids, one of which carries the toxin-coding genes, with four occurrences of the distinct *orf1*-gap-*orf2* organization. Moreover, this plasmid is extremely rich in mobile genetic elements, unlike its two coresidents. This highlights the important underlying evolutionary traits between toxin-carrying plasmids and the adaptation of a B. thuringiensis strain to its environment and insect host spectrum.

## Abstract (precis)

Bacillus thuringiensis, a soil entomopathogenic bacterium, is at the base of many sustainable eco-friendly bioinsecticides. Hence stems the need to continually characterize insecticidal toxins.

## INTRODUCTION

Bacillus cereus sensu lato, a diverse group of spore-forming Gram-positive bacteria, contains nine closely related species with a wide pathogenicity spectrum but whose exact phylogeny remains a matter of debate (1–3). Bacillus thuringiensis, a soil-dwelling bacterium, is one of the best-studied members of this group. In fact, B. thuringiensis strains are commercialized and used worldwide as biopesticides in an effort to replace harmful chemical insecticides in the fight against disease-carrying and phytopathogenic insects. Their entomopathogenic capacities are due to a parasporal crystal, formed during sporulation and consisting of δ-endotoxins (or Cry), sometimes associated with Cyt cytotoxins. This crystal is solubilized after ingestion by the alkaline environment of the target larva midgut (4).

Cry toxins, activated by proteases, bind to specific receptors localized in the apical microvilli of insect midgut cells. This is followed by an oligomerization of the toxins and their insertion into the apical membrane, leading to the formation of pores that cause intestinal cell lysis and consequently the death of the insect larvae (5). So far, the B. thuringiensis toxin database, set up by Crickmore et al. (6) (https://www.bpprc.org/ [7]) encompasses 73 families of three-domain (3D) Cry toxins. Whereas domain I presents an α-helical structure and is involved in toxin oligomerization and membrane insertion thus pore formation, domains II and III have β-prism and β-sheet structures, respectively, and have been found to mediate recognition and interaction with insect gut protein receptors (5, 8, 9). A key feature of all Cry toxins is their receptor specificity. The combination of Cry toxins produced by a particular strain determines its host spectrum (10). In contrast, Cyt toxins are cytolytic endotoxins that act via nonspecific detergent-like (11) or pore-forming (12) models. Remarkably, Cyt toxins may show significant homology with proteins produced by distant microorganisms, such as volvatoxin A2, a heat-labile cardiotoxin produced by the straw mushroom Volvariella volvacea (13), as well as other proteins found in pathogenic microorganisms (e.g., Clostridium kluyveri or Streptomyces venezuelae) (14).

The combination of various toxins in the same crystal leads to synergistic interactions among Cry toxins or between Cry and Cyt toxins when both are present. The synergy can increase toxicity and play against the emergence of resistant insect populations (15). This has been observed, for instance, for the combination of Cry2A and Cry4B (16) and for that of Cry21Fa1 and Cry21Ha1 (17). Similar observations have been made in the case of B. thuringiensis serovar (sv.) israelensis, in which Cyt1A helps Cry4 toxins to overcome resistance of Culex quinquefasciatus larvae (18). Cyt1A was also found to act as a receptor and anchor for Cry11Aa to increase its toxicity to Aedes aegypti larvae (19), and Cyt1A and Cyt2A were shown to synergize Cry4B toxins, at a ratio as low as 1% in the mix (20, 21).

B. thuringiensis sv. israelensis is an example of a highly effective combination of toxins. It is the reference for antidipteran activity, to which no resistance has been observed in the field despite its long usage as a biocontrol agent due to the mix of Cry toxins as well as the presence of the Cyt toxins (22). Its crystal is composed of Cry4Aa, Cry4Ba, Cry10Aa, Cry11Aa, Cyt1Aa, and Cyt2Ba, with Cyt being the major component. These toxin-encoding genes are located on the 128-kb toxin-carrying plasmid pBtoxis (23).

Plasmids are part of the repertoire of mobile genetic elements (MGEs) of each strain. Some of these extrachromosomal entities can be conjugative (24), mobilizable, or of prophage-like nature (25). Another group of MGEs are transposable elements (TEs), which can move from one location to another within the same genome. They include insertion sequences (IS), with a simple organization of two inverted repeats flanking a transposase-coding gene (26), or more complex elements, such as class II TEs (27). TEs also include composite transposons, with two IS flanking one or more passenger genes (28). In addition, MGEs comprise group II introns, which have a complex secondary RNA structure (29), and Bacillus cereus repeats small DNA fragments found only in the B. cereus group (30). In this group, the occurrence and distribution of MGE types and families vary greatly among its members, as has recently been shown (31).

Plasmids, more specifically those carrying toxin genetic determinants, are key elements of the ecology and adaptation of B. cereus sensu lato species, especially in the case of B. thuringiensis, whose plasmid percentage of the genome is 2.66 times higher than that of its closest relative, B. cereus sensu stricto (31). Toxin-carrying plasmids form up to 30% of the B. thuringiensis plasmid pool, and toxin-coding genes are often associated with transposable elements (TEs), such as IS or class II transposable elements (32–34).

B. thuringiensis is the basis of many efficient biopesticidal products available on the market (e.g., Vectobac and Monterey *B.t.*). Nevertheless, the scientific community is always on the lookout for new active B. thuringiensis strains producing novel Cry toxins.

In this study, we characterized a new antidipteran B. thuringiensis strain, isolated from Lebanese soil, whose spore-crystal mixture lacks Cyt toxins and displays a novel *in vivo* killing profile. We have also found that this H3 strain harbors a plethora of mobile genetic elements, often associated with the entomotoxin genetic determinants.

## RESULTS

### Bacterial strains and bioassays.

**FIG 1:**
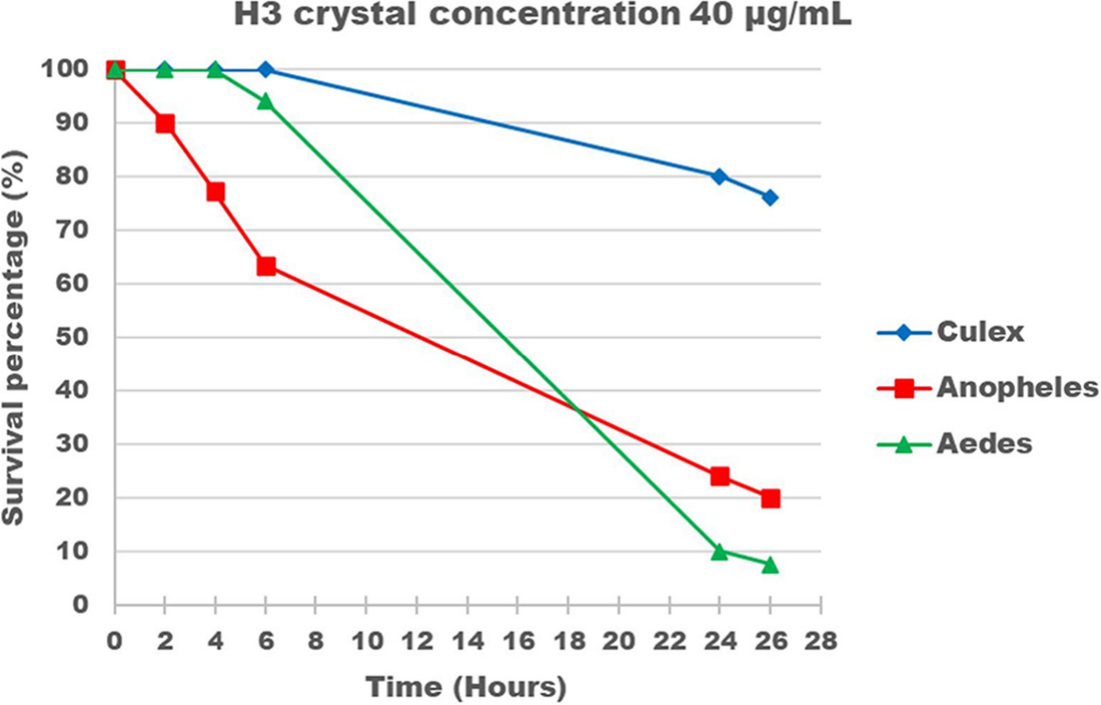
Toxicity analysis of the spore-crystal mixture of B. thuringiensis strain H3 on third-instar larvae of *A. albopictus*, A. gambiae, and *C. pipiens*. Graphs show the percentage of survivors through time for a crystal-spore mixture concentration of 40 µg/ml.

B. thuringiensis strain H3 was isolated from a soil sample from the region of Harissa (Lebanon). This strain produces a round parasporal crystal. In an effort to find new antidipteran B. thuringiensis strains, the H3 crystal-spore mixture was tested against third-instar larvae of Aedes albopictus, Culex pipiens, and Anopheles gambiae. Initial survival tests were performed using five crystal proteins concentrations (5, 10, 20, 30, and 40 μg/ml). H3 was noticeably less toxic to *Culex* than to *Anopheles* and *Aedes* (Table 1 and Fig. 1), with 50% lethal concentration (LC_50_) values after 24 h of 221 ± 98, 15.8 ± 3.43, and 7.6 ± 0.88 µg/ml, respectively. As shown in Fig. 1, for a δ-endotoxin concentration of 40 µg/ml, the survival rate was ca. 80% for *C. pipiens* larvae after 24 h, compared to 24% and 10% for A. gambiae and *A. albopictus*, respectively. As for the reference strain B. thuringiensis sv. israelensis AM65-52, the LC_50_ values, calculated 2 h 30 min postinfection for *Culex*, 3 h 30 min postinfection for *Anopheles*, and 5 h postinfection for *Aedes*, were 7.4 ± 0.11, 33.36 ± 0.5, and 8.39 ± 0.09 ng/ml, respectively (35).

**TABLE 1:** Larvicidal activities of B. thuringiensis strains AM65-52 and H3 on third-instar larvae of *A. albopictus*, A. gambiae, and *C. pipiens*

Organism	B. thuringiensis strain H3		B. thuringiensis sv. israelensis strain AM65-52	
	Time postinfection	LC_50_, mean ± SD (µg/ml)	Time postinfection	LC_50_, mean ± SD (×10^−4^ µg/ml)
*A. albopictus*	24 h	7.6 ± 0.88	5 h	83.9 ± 0.9
A. gambiae	24 h	15.8 ± 3.43	3 h 30 min	336.5 ± 5
*C. pipiens*	24 h	221.8 ± 98	2 h 30 min	74 ± 1.1

While AM65-52 showed the highest activity against *Culex*, this was not the case for H3, which was most toxic to *Aedes* larvae, on which additional bioassays were conducted. Third-instar *A. albopictus* larvae were exposed to various combinations of H3 and AM65-52 spore-crystal mixtures at a final concentration of 40 µg/ml of toxin. Subsequently, the 50% lethal time (LT_50_) of the combination and that AM65-52 spore-crystal mixture alone were compared. The combination of H3 and AM65-52 resulted in a reduction of the AM65-52 LT_50_ via a potential additive effect for all tested combinations with varied proportions of the spore-crystal mixtures (see Fig. S1 in the supplemental material). For instance, in a comparison of the 90:10 ratio of H3 to AM65-52 to AM65-52 alone at a 10% ratio of the total 40-µg/ml concentration (i.e., at 4 µg/ml), the observed LT_50_ was ca. 60 instead of 80 min.

**FIG 2:**
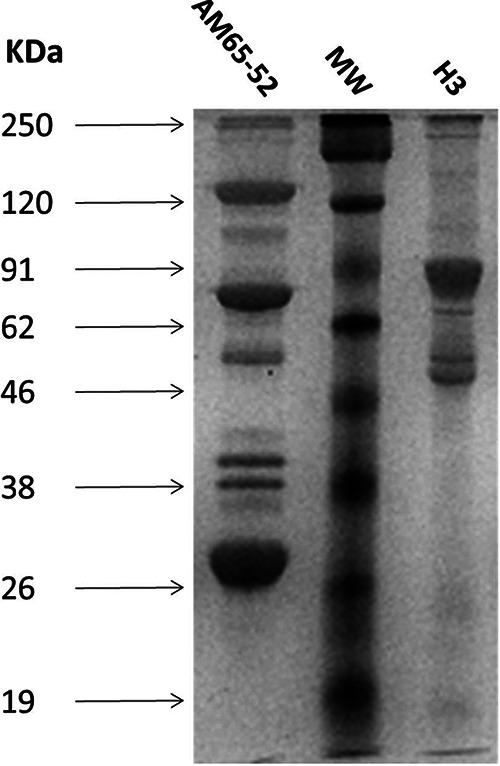
Washed crystal protein profile on 12% SDS-PAGE of H3 and the reference B. thuringiensis sv. israelensis strain AM65-52. MW, molecular weight markers; sizes are indicated on the left.

The Cry toxin composition of strain H3 was then assessed for its protein profile, which was compared to that of AM65-52. The two strains did not share any protein bands, reflecting their different crystal compositions (Fig. 2). Moreover, PCR screening of genes encoding known antidipteran Cry proteins (Cry4A/B, Cry10, and Cry11) was negative with only a partial segment of the 5′ end of the *cry4B* gene amplified with Dip2A-Dip2B primers (36). Taken together, these results strongly suggested that strain H3 produces new antidipteran toxins. PCR with genes encoding Cyt toxins (Cyt1A and Cyt2B) was also negative, meaning that H3, contrary to classic antidipteran strains, does not produce those Cyt toxins. Moreover, washed, solubilized, and filtered crystals of H3 and AM65-52 were tested on 5% sheep blood agar. While AM65-52 crystals showed a clear halo where the blood cells were lysed, H3 crystals did not (Fig. S2).

### Whole-genome sequencing of strain H3.

In order to characterize the potentially new Cry toxins produced by strain H3, a whole-genome sequencing (WGS) approach was performed using PacBio, with Illumina MiSeq polishing. The assembled genome of B. thuringiensis H3 consists of four replicons: a chromosome of 5,487,336 bp and three large plasmids: pH3-552 (552,228 bp), pH3-180 (180,731 bp), and pH3-101 (101,260 bp). Sequences of the 6,321,555-bp genome were deposited in NCBI genome database under the BioProject identifier (ID) PRJNA611745 and GenBank accession numbers CP052061 to CP052064. Sequence information for each replicon is shown in Table 2.

**TABLE 2:** Genomic features of B. thuringiensis strain H3

Replicon	Length (bp)	G+C content (%)	No. of CDSs
Chromosome	5,487,336	35.4	5,621
pH3-552	552,228	32.9	430
pH3-180	180,731	34.1	179
pH3-101	101,260	32.5	94

**FIG 3:**
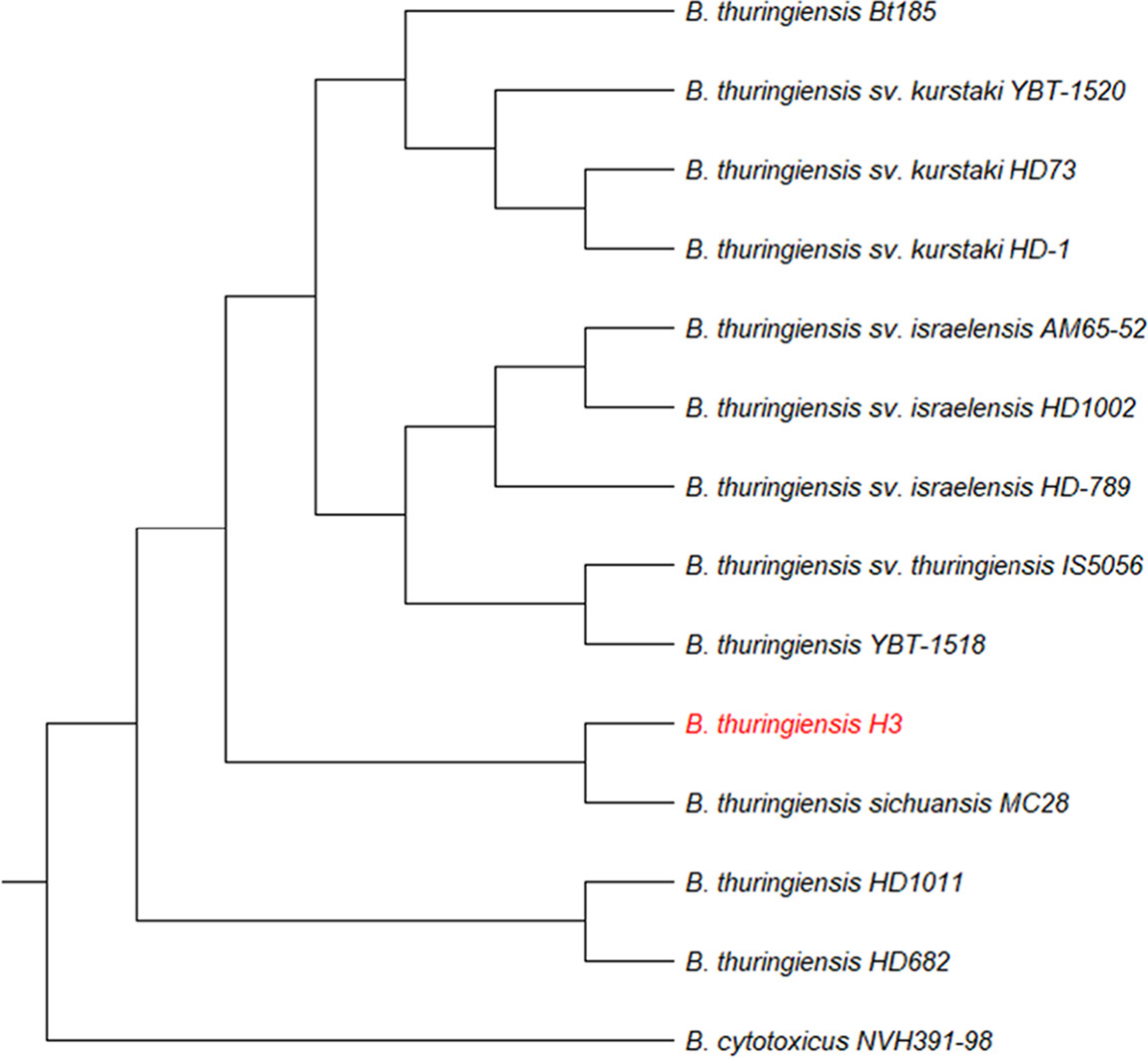
SNP-based relationship dendrogram of several B. thuringiensis strains, with *B. cytotoxicus* NVH391-98 as an outgroup. Chromosomic DNA alignment and SNP extraction were done using progressiveMauve (37). The dendrogram was drawn using MEGA-X v10.0.5 (76).

The H3 chromosome was aligned with the chromosomes of 12 B. thuringiensis strains and one Bacillus cytotoxicus strain using progressiveMauve (37). Following this alignment, single nucleotide polymorphisms (SNPs) were extracted and compared between strains. On the basis of SNP divergence between the strains, a maximum-likelihood relationship dendrogram was constructed. This analysis showed that H3 is closely related to B. thuringiensis sv. sichuansis strain MC28 (GenBank accession no. CP003687.1 [38]) as shown by the relationship analysis (Fig. 3). Both strains showed antidipteran activity. As for H3 plasmids, they amount to ca. 12% of the H3 genome.

### Novel insecticidal toxins.

**FIG 4:**
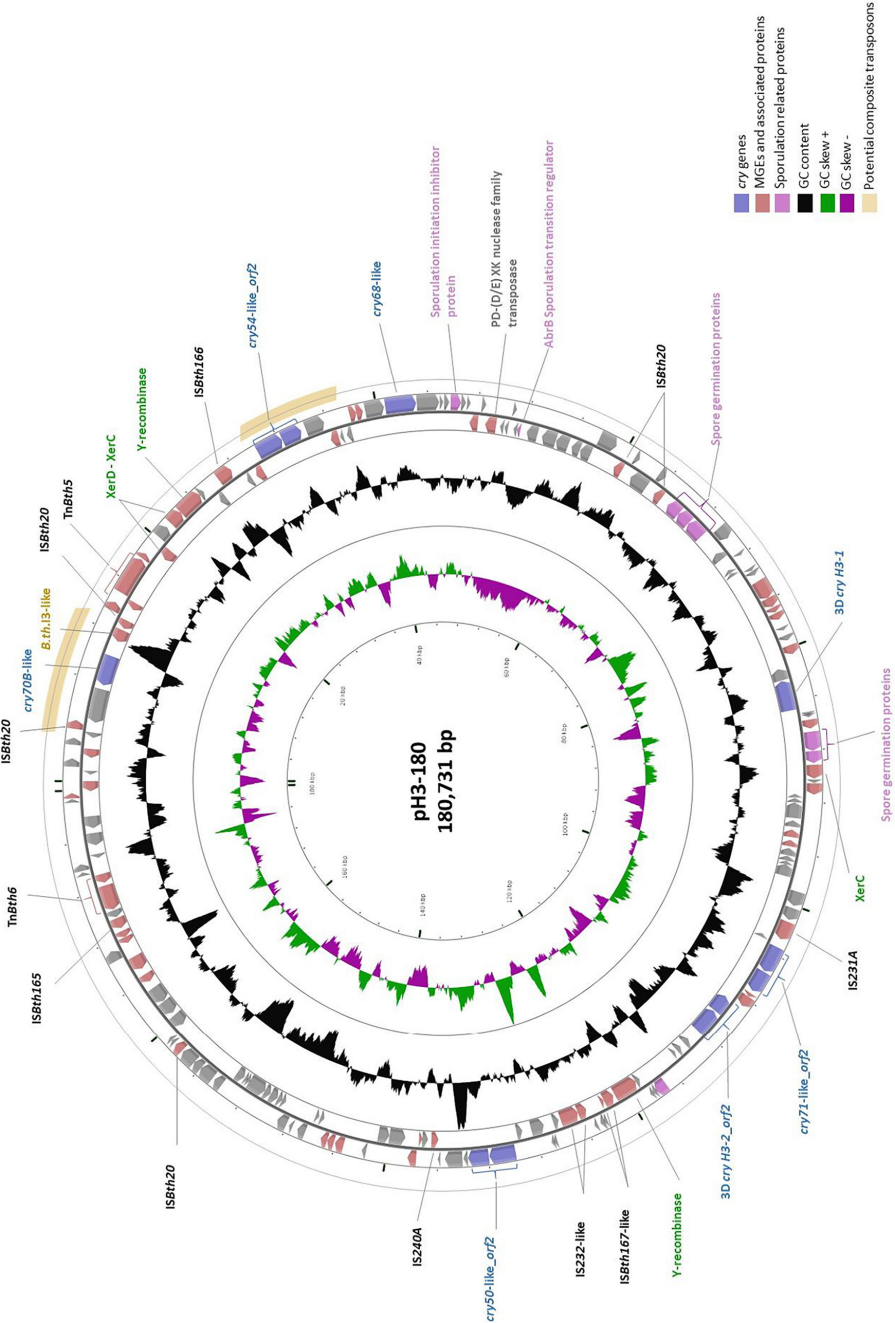
Circular map of B. thuringiensis strain H3 toxin-carrying plasmid pH3-180. The block arrows in the outer circles indicate the predicted open reading frames (ORFs) in their direction of transcription, with or without functional annotation or relevant homologues. The black circle represents the GC content plotted using a sliding window, as the deviation from the average GC content of the entire sequence. The green/magenta circles represent the GC skew calculated, using a sliding window, as (G−C)/(G+C) and plotted as the deviation from the average GC skew of the entire sequence. ORFs encoding Cry toxins, transposable elements with their associated ORFs, and sporulation-related proteins are highlighted by blue, dark pink, and fuchsia block arrows, respectively. The composite transposons containing *cry* genes are indicated by gold arcs on the outermost circle.

Eleven putative insecticidal *cry* genes were identified on pH3-180 (Fig. 4) by Prokka and BtToxin_scanner annotations. Encoded proteins were compared by BLAST.P (39) to the NCBI nonredundant protein database and checked for the δ-endotoxin conserved domains by CDART (40). No *cyt* genes were found in the entire H3 genome. As shown in Table 3, seven H3 Cry proteins contain at least two conserved key insecticidal domains, one or two N-terminal endotoxin domains, and the C-terminal carbohydrate binding modules. Parts of the endotoxin domains are cleaved and eliminated during toxin activation to subsequently allow membrane insertion and pore formation. As for the carbohydrate binding module, it is implicated in toxin-receptor interactions (41).

**TABLE 3:** Insecticidal Cry proteins produced by B. thuringiensis strain H3^*a*^

Assigned name^*b*^	Transcription orientation	Length in amino acids (predicted mol wt in kDa)	Possible operon (intergenic distance in bp)^*c*^	NCBI (CDART) hits^*d*^
Cry70B-like protein	−	808 (90.89)		(1) Endotoxin_N (2) Carbohydrate binding module
Cry54-like protein	+	698 (79.18)	Yes (21)	(1) Endotoxin_N (2) Endotoxin_M (3) Carbohydrate binding module
**ORF2**	**+**	**535 (60.8)**		**Insecticidal δ-endotoxin CryIA(c) domain 5**
Cry68-like protein	+	804 (89.7)		(1) Endotoxin_N (2) Endotoxin_M (3) Carbohydrate binding module (4) Ricin
3D Cry H3-1^*e*^	−	818 (91.24)		(1) Endotoxin_N (2) Endotoxin_M (3) Carbohydrate binding module (4) Ricin
Cry71-like protein	+	688 (78.19)	Yes (68)	(1) Endotoxin_N (2) Endotoxin_M (3) Carbohydrate binding module
**ORF2**	**+**	**563 (64.5)**		**Insecticidal δ-endotoxin CryIA(c) domain 5**
**ORF2**	**−**	**486 (55.14)**	**Yes (68)**	**Insecticidal δ-endotoxin CryIA(c) domain 5**
3D Cry H3-2^*e*^	−	688 (78.76)		(1) Endotoxin_N (2) Endotoxin_M (3) Carbohydrate binding module
Cry50-like protein	+	688 (78.21)	Yes (31)	(1) Endotoxin_N (2) Endotoxin_M (3) Carbohydrate binding module
**ORF2**	**+**	**537 (60.93)**		**Insecticidal δ-endotoxin CryIA(c) domain 5**

a Proteins with an ORF1-gap-ORF2 organization are highlighted by gray shading.

b Names were assigned after comparison with the B. thuringiensis nomenclature database.

c Between the stop codon of the first open reading frame and the start codon of the second one.

d The order of the conserved domains in the protein in an N- to C-terminal orientation.

e Three-domain Cry toxin.

Four other genes (all designated ORF2) were also annotated as potentially associated with an insecticidal function. The proteins they encode contain a newly recognized crystallization domain following CDART analysis (40) and present a high sequence similarity to each other and to the C-terminal part of Cry4-type proteins. In comparison to Cry4Ba5 from B. thuringiensis sv. israelensis plasmid pBtoxis, the conserved area is located from amino acid position 656 to the end of the protein (Fig. S3). This high sequence similarity strongly suggests a conserved function with the crystallization domain of Cry4-type proteins, which is essential for their inclusion in the parasporal crystal (42). Interestingly, the genes encoding these proteins are located downstream of those encompassing conserved domains, with a short intergenic distance (max. 68 nt), as indicated in Table 3. Moreover, the nucleotide sequences of these gaps are almost identical, especially just before the start codon of *orf2*, where the consensus sequence is T-aaAAAGGTtGTGAaTcaT (upper- and lowercase letters indicate a more- or less-conserved nucleotide in the gap sequence). This sequence is also found in full-length *cry4*-type genes, upstream of the conserved C-terminal coding part. This is in line with a previous study that suggested a common origin for these *orf2* coding genes: a truncated C-terminal part of a *cry4*-type gene that resulted from a rearrangement leading to an *orf1*-gap-*orf2* organization (43).

In order to confirm that H3 actually expresses these *cry* genes, a detailed characterization of the protein content of the whole crystal was conducted using nano-liquid chromatography-tandem mass spectrometry (nano-LC MS/MS) (44). The resulting peptides were used to identify the various proteins by homology search in the UniProt B. thuringiensis database. These peptides were cross-checked with the 11 Cry proteins predicted by WGS. The results confirmed the presence of the 11 potential proteins in H3 crystal: 7 with the conserved insecticidal domains, ranging in size between 78.19 and 91.24 kDa, and 4 with a newly identified crystallization domain, ranging between 55 and 65 kDa (Fig. 2 and Table S3).

Each of the seven newly identified Cry proteins with conserved insecticidal domains was compared to entries from the B. thuringiensis nomenclature database (7), following which a name was designated (Table 3). Proteins showing less than 45% sequence similarities with annotated Cry proteins from the B. thuringiensis nomenclature database, hence not passing the rank 1 threshold, were assigned as three-domain Cry toxins (3D Cry H3-1 and H3-2). Four other proteins showing sequence identity between 45 and 78% to annotated Cry toxins were consequently labeled Cry50-, Cry54-, Cry68-, and Cry71-like proteins. The last of the seven proteins, passing the rank2 threshold of 78% sequence identity with Cry70B, was named Cry70B-like protein.

### pH3-180, the unusual mosquitocidal plasmid.

pH3-180 is the only toxin-carrying plasmid of B. thuringiensis H3. In addition to *cry* genes, pH3-180 holds 35 elements belonging to 10 IS and class II transposable element families (Table S1) and one group II intron (Fig. 4). Interestingly, ca. 22% of pH3-180 is occupied by IS and Tn*3*-like elements. Some IS elements, such as the IS*240A*-like element from the IS*6* family, are also found on B. thuringiensis sv. israelensis 128-kb toxin plasmid pBtoxis (45), while others, like IS*Bth166* from the IS*110* family, are absent from pBtoxis.

The most abundant element on pH3-180 is the IS*Bth20*-like element (IS*6* family), present in 12 copies. The abundance of this element and its distribution on pH3-180 increase the possibility of forming composite transposons, with two IS*Bth20*-like elements flanking one or more passenger genes. Even though composite transposons are not limited by size, those with the closest bordering IS*Bth20*-like elements and a size under 10 kb are shown in Table S2. However, the two most noteworthy are those where IS*Bth20*-like elements flank toxin-coding genes, as highlighted in Fig. 4 by golden arcs on the outermost circle. Such is the case for the first transposon, with *cry70B*-like, the *B.th.*I3-like group II intron, and a Tn*4430*-like transposase-coding gene, flanked by two iso-IS*Bth20* elements in opposite orientations. The second composite transposon includes two iso-IS*Bth20* elements in the same orientation flanking the *cry54*-like gene and its downstream *orf2*, as well as a phospholipase C-coding gene.pH3-180 also holds a transposase-coding gene from a potentially new family, identified as a PD-(D/E)XK nuclease family transposase. This superfamily encompasses proteins with diverse functional niches, such as DNA restriction, tRNA splicing, and DNA transposition (46). Although this protein superfamily remains somewhat unexplored in the transposition research field, PD-(D/E)XK putative transposase has been located in a number of microorganisms, including archaeal and bacterial strains (47).

Two new class II transposable elements were identified on pH3-180 and were designated Tn*Bth5* and Tn*Bth6*. The former has a characteristic Tn*3* family organization with 49- and 50-bp terminal inverted repeats flanking transposase- and resolvase-coding genes in opposite orientations (Fig. S4A). Interestingly, a second and identical copy of Tn*Bth5* is present on the 100-kb plasmid pH3-101. As for Tn*Bth6*, it holds three coding DNA sequences (CDSs) in the same orientation, one of which is a resolvase and the other two seem to belong to a disrupted class II transposase (Fig. S4B). These CDSs are flanked by 48- and 50-bp-long inverted repeats.

Other TEs include the aforementioned *B.th*.I3-like group II intron, originally found in B. thuringiensis sv. israelensis strain ATCC 35646 within a gene coding a reverse transcriptase (48). In the case of strain H3, this element is missing ca. 500 bp at the 3′ end in comparison with *B.th.*I3 and carries a 335-amino-acid group II intron reverse transcriptase/maturase. The *B.th.*I3-like intron is located between an insecticidal toxin-coding gene and an IS*Bth20*-like element, as mentioned above, without disrupting any CDS. In addition to these MGEs, seven tyrosine recombinase/integrase-coding genes are distributed on pH3-180. They may function independently or in association with other pH3-180 mobile elements.

### Other interesting features.

In addition to toxin genes and mobile elements, pH3-180 carries two operons containing three and two spore germination protein-encoding genes (Fig. 4). Moreover, pH3-180 holds two copies of a gene encoding the sporulation initiation inhibitor protein Soj (49) and one *abrB* gene, whose function is to control the transition from vegetative state to sporulation (50). Interestingly, a similar situation is observed for pH3-552, the large coresident plasmid, which harbors several genetic loci related to spore formation or germination.

Beside the Cry toxins, several noteworthy features are associated with strain H3 and could intervene in its interaction with surrounding environment. Many of these features are carried by the 552-kb megaplasmid pH3-552. These include several putative two-component systems and six ABC transporter systems. Concerning antibiotics, pH3-552 holds potential genetic determinants for fosfomycin resistance and bacitracin and gramicidin S synthesis. Strain H3 also carries genes encoding hydrolytic enzymes, also referred to as cell wall-degrading enzymes: cellulase, on pH3-552, and chitinase, on pH3-552 and the chromosome. Two other types of bioactive molecules, siderophores and lassopeptides, potentially produced by strain H3 are encoded on the chromosome.

## DISCUSSION

Diptera, especially mosquitoes, serve as vectors for the transmission of many animal diseases, such as malaria, dengue, or yellow fever. So far, the commonly used method to fight these vector-borne diseases is to kill the mosquitoes with synthetic chemical insecticides. However, biopesticides, more particularly those based on B. thuringiensis, are slowly but surely taking over the insecticide market. Nonetheless, the need for prevention of potential emergence of resistant strains due to the prolonged use of the same combination of toxins in the field has motivated the search and characterization of novel insecticidal strains and toxins. In this study, a new B. thuringiensis strain, H3, isolated from Lebanese soil and active against dipteran larvae was characterized for both its insecticidal potential and genomic composition.

Strain H3 is toxic for third-instar larvae of *A. albopictus*, *C. pipiens*, and A. gambiae but at a higher dose than the reference B. thuringiensis israelensis serovar. However, H3 crystal lacks Cyt toxins, which could explain the observed higher dose required for its toxicity. Interesting results were also obtained with the combinations of H3 and AM65-52 spore-crystal mixture, following which the LT_50_ of AM65-52 was reduced. This raised a question regarding the nature of the interaction between the crystal toxins of AM65-52 and H3, which can be either an additive effect or a synergistic interaction. If indeed the observed effect stems from synergy between the toxins of the two strains, a second question ensues, which evokes the identity of the main actors in this interaction from the two strains’ crystal proteins. B. thuringiensis sv. israelensis Cyt toxins are a major component of the crystal and have a key role in its toxicity through their individual and synergistic activities with Cry toxins (19–21). In fact, thanks to their capacity to recognize and interact with the phospholipids of the insect midgut cells, Cyt toxins can act as receptors for Cry toxins in case of a shift or to simply increase the number of receptor-toxin interactions. This is why Cyt toxins, particularly Cyt1A, are well-known key players in the described synergism in many cases with several B. thuringiensis Cry toxins (51–53). Nonetheless, the possibility of synergistic interactions between the Cry toxins of the two strains cannot be excluded. Hence, three aspects need to be further explored in future experiments, which will consist of detailed synergistic tests between (i) Cyt1Aa and H3 crystal proteins, (ii) Cyt1Aa and individually cloned H3 crystal proteins, and (iii) paired combinations of individually cloned H3 and AM65-52 crystal proteins (i.e., Cry4Aa with Cry70B-like protein and Cry10Aa with Cry68-like protein).

Moreover, B. thuringiensis sv. israelensis is known to be more toxic to *Culex* than to *Aedes* and *Anopheles* larvae (35), in contrast to H3. Since the dose- and time-dependent killing profiles are indications of activity via specific receptors in the insect midgut, this led us to believe that H3 toxins kill the larvae in a different way than what was described for B. thuringiensis sv. israelensis. In agreement with the observed difference in toxicities between H3 and the reference B. thuringiensis sv. israelensis AM652-52, the crystal protein profile of the former is also completely different from that of the latter. In order to get more insight into the genetics and genomics of H3 entomotoxins, a whole-genome sequencing approach was used. The strain H3 chromosome appeared to be closely related to the chromosome of B. thuringiensis sv. sichuansis MC28, which also displays antidipteran activity (54). Moreover, H3 holds three new large plasmids, accounting for almost 12% of its genome, hence, in size, a higher-than-average plasmid percentage (11.2%) but a lower plasmid number per strain of B. thuringiensis (6.4 plasmids per stain [31]).pH3-180, the toxin-carrying plasmid, harbors 11 putative *cry* genes coding for potential parasporal crystal toxins. Seven of these proteins hold conserved insecticidal domains, of which five were placed in specific three-domain Cry toxins families displaying mosquitocidal activity (55) (Cry50, Cry54, Cry68, Cry70, and Cry71). The remaining two proteins (3D Cry H3-1 and H3-2) did not show enough protein sequence identity with any known Cry family but contained conserved insecticidal domains found in three-domain Cry toxins (Table 3). Even though these seven proteins have the necessary domains for insecticidal activity, it is unclear which one(s) is responsible for H3 antidipteran activity, since these toxins are new and not characterized yet. Therefore, a more in-depth investigation is needed for the assessment of the individual toxicity of each protein and the identification of its target receptors in the insect midgut.

As for the remaining four, they are designated ORF2 proteins, thought to be indispensable for correct expression, folding, and crystallization of the Cry toxin to which they are associated, as shown by previous studies. Barboza-Corona et al. have indeed shown that in the case of the *cry19A* operon, ORF2 enhances the synthesis and crystallization of Cry19A by functioning as a C-terminal crystallization domain (56). This was also demonstrated later on for Cry30 (57), Cry5B (58), and Cry65A (59). Another important *orf1*-gap-*orf2* operon organization is that of B. thuringiensis sv. israelensis Cry10Aa protein, which requires ORF2 for correct crystallization, in contrast to Cry11Aa, which benefits from the presence of the 20-kDa helper protein p20 (43, 60). A 2015 study by Peng et al. suggested that this could be a new evolutionary strategy for B. thuringiensis Cry proteins and could explain why some Cry proteins cannot form crystals when expressed alone in an acrystalliferous strain (59). Interestingly, though, in the case of H3, four toxin genes adopt the *orf1-gap-orf2* organization.

pH3-180 is also quite unusual for the abundance of TEs, including more than 35 IS and class II elements, a group II intron, and several potential composite transposons. For comparison, the large coresident pH3-552 plasmid (552 kb) holds only eight IS elements, belonging to three families (IS*3*, IS*200*/IS*605*, and IS*4*). Toxin-carrying plasmids in B. thuringiensis are no strangers to TEs, since they hold a good proportion of the total plasmidial IS, Tn*3*-like elements, and group II introns (31). As previously mentioned, several TEs were found to be associated with toxin-coding genes (e.g., IS*231* [61]) and, when active, to shuffle the *cry* genes within the bacterial genome and also among strains in the case of conjugation/mobilization events. Of notice, however, is the 22% of pH3-180 occupied by TEs, a figure much higher than the average of 7.46% for toxin-carrying plasmids (31) and the 3.8% for the B. thuringiensis sv. israelensis pBtoxis plasmid (45). This is also much higher than the IS/Tn*3*-like element proportions of pH3-101 (5.9%) and pH3-552 (1.9%).

TE prevalence in toxin-carrying plasmids compared to other plasmids may underline an evolutionary adaptation mechanism, due to their contribution to the diversity of B. thuringiensis virulence and its insect host spectrum. Not only are toxin-carrying plasmids important for the ecological function of B. thuringiensis, but also they play key roles in other cellular functions, such as sporulation/germination. Some studies have suggested that these plasmids are the defining genomic entity for the recognition of a strain as a member of the B. thuringiensis species (62), although in some rare cases, *cry* genes were found on the chromosome, surrounded by IS elements, a possible reason for their mobility from plasmid to chromosome (63).

Another aspect of H3 toxicity brought to light by the whole-genome sequencing is a group of bioactive molecules possibly produced by H3. Many of these molecules are encoded by pH3-552. They include bacitracin (64) and gramicidin S (65), cyclic nonribosomal peptides mostly active against Gram-positive bacteria. In addition, H3, like other B. thuringiensis strains, potentially produces chitinase, an enzyme with great potential as an agent for biocontrol of pathogenic fungi (66) and as an added value to the insecticidal activity thanks to the degradation of chitin laminating the intestinal peritrophic membrane (67). Copies of the chitinase gene are found on the H3 chromosome as well as pH3-552.

The H3 chromosome also carries clusters for two other potential bioactive molecules: siderophores, iron-scavenging molecules that may act as biocontrol agents by chelating iron and reducing its bioavailability (64), and lassopeptides, ribosomally synthesized and posttranslationally modified peptides (RiPPs) with a unique 3-dimensional structure and a wide spectrum of antimicrobial and analgesic activities (68).

In conclusion, H3 is an interesting B. thuringiensis strain with a new plasmidial content, including a wide MGE repertoire within pH3-180, the toxin-carrying plasmid. The novelty of H3 Cry proteins, with their different killing profile, is encouraging for their potential use in the field as coformulants to the classic antidipteran strains. Nevertheless, these findings also raise several issues that pave the way for future studies, such as the individual toxicity of each of these new toxins and the identification of the receptors to which they bind, the role of ORF2 in the expression and correct folding of its associated protein, and, finally, the potential activity and effect of TEs within the toxin-carrying plasmid pH3-180.

## MATERIALS AND METHODS

### Bacterial strains.

B. thuringiensis sv. israelensis AM65-52, isolated from the commercial sample (Vectobac; Sumi- tomo), was our reference strain. It was kindly provided by Christina Nielsen-Leroux from the GME laboratory of the National Institute for Agronomical Research (INRA, Jouy-en-Josas, France). Strain H3 was isolated from Lebanese soil and selected from a group of strains tested against third-instar mosquito larvae of Aedes albopictus, Culex pipiens, and Anopheles gambiae and screened by PCR for genes encoding known antidipteran Cry (Cry4A/B, Cry10, and Cry11) and Cyt toxins. Microorganisms and primers used in this study are shown in Tables 4 and 5.

**TABLE 4:** Bacterial and mosquito strains used in this study

Organism	Reference or source
Bacteria	
B. thuringiensis H3	This study
B. thuringiensis sv. israelensis AM65-52	VectoBac, provided by Christina Nielsen-Leroux, GME Laboratory, National Institute for Agronomical Research (INRAE, Jouy-en-Josas, France)

Mosquitoes	
Aedes albopictus	Biology department, American University of Beirut (Zakaria Kambris)
Culex pipiens	Biology department, American University of Beirut (Zakaria Kambris)
Anopheles gambiae	Biology department, American University of Beirut (Mike Osta)

**TABLE 5:** List of primers used in this study

Primer pair	Sequences, forward/reverse (5′→ 3′)	Target gene(s)	Reference
Dip1A/-1B	CAAGCCGCAAATCTTGTGGA/ATGGCTTGTTTCGCTACATC	*cry4A* and *cry4B*	36
Dip2A/-2B	GGTGCTTCCTATTCTTTGGC/TGACCAGGTCCCTTGATTAC	*cry4B*	36
Cry10F/-R	TCAATGCTCCATCCAATG/CTTGTATAGGCCTTCCTCCG	*cry10*	80
Cry11F/-R	CGCTTACAGGATGGATAGG/GCTGAAACGGCACGAATATAATA	*cry11A* and *cry11B*	80
Cyt1F/-R	CCTCAATCAACAGCAAGGGTTATT/TGCAAACAGGACATTGTATGTGTAATT	*cyt1A* and *cyt1B*	80
Cyt2F/-R	ATTACAAATTGCAAATGGTATTCC/TTTCAACATCCACAGTAATTTCAAATGC	*cyt2A*, *cyt2B*, and *cyt2C*	80

### Crystal protein extraction and SDS-PAGE analysis.

B. thuringiensis strains were grown on solid T3 medium (69) for 72 h at 30°C. Spores and crystals were collected and washed twice with 1 M sodium chloride solution and six times with cold sterile water. The spore-crystal mixtures were then solubilized in 50 mM sodium hydroxide (NaOH) buffer at 30°C (70). This was followed by centrifugation to eliminate remaining cellular debris and spores. Solubilized crystal proteins were then quantified using Bradford reagent (Bio-Rad protein assay, catalog no. 500-0006 [71]). The proteins were separated by 12% SDS-PAGE and stained with Coomassie brilliant blue for profiling.

### Bioassays.

Bioassays were conducted on third-instar larvae of *A. albopictus*, *C. pipiens*, and A. gambiae, reared in the biology department of the American University of Beirut at 27°C and with 80% relative humidity and a 12-h:12-h dark:light period. A. gambiae larvae were kindly provided by Mike Osta. Bioassays were performed in three biological replicates, each time with experimental triplicates on 14 larvae in 15 ml of dechlorinated water. For B. thuringiensis sv. israelensis strain AM65-52, concentrations of crystal proteins within the crystal-spore mixture were aligned with known B. thuringiensis sv. israelensis crystal protein concentrations. As for H3, its spore-crystal mixture was tested at a range of crystal protein concentrations (5, 10, 20, 30 and 40 μg/ml) and then set at 40 µg/ml for comparison of H3 activities on the different insect larvae. Treated larvae were housed at 27°C and examined after 2, 4, 6, 24, and 26 h. Additional bioassays were conducted on third-instar *A. albopictus* larvae by combining spore-crystal mixtures of H3 and the reference strain B. thuringiensis sv. israelensis AM65-52 with five H3/AM65-52 ratios (95:5, 90:10, 80:20, 70:30, and 50:50), while maintaining a total concentration of 40 µg/ml. The combination of H3 and AM65-52 crystal-spore mixtures was compared to that of H3 and AM65-52 alone, by evaluating the 50% lethal time (LT_50_) of the larvae.

Survival was determined by counting dead larvae over 160 min with 20-min intervals.

### Plasmid DNA profiling.

Large plasmids, hard to visualize using standard techniques, were extracted following the procedure originally described by Andrup et al. (72) and recently adapted by Gillis et al. (25). Plasmids were visualized on 0.5% SeaKem GTG agarose gel after 24 h of migration at 4^°^C and 80 V.

### Whole-genome sequencing.

Genomic DNA was extracted using the Wizard genomic DNA purification kit from Promega. Sequencing was done by PacBio on library DNA with a 7-kb size cutoff. Resulting sequences were polished by alignment with Illumina MiSeq data using bbmap (73). The final sequence was circularized by PCR, annotated by Prokka (74), and checked for completeness by BUSCO (75).

Phylogenetic relationships between H3 and 12 B. thuringiensis strains were assessed following alignment of chromosomes and extraction of single nucleotide polymorphisms (SNPs) with progressiveMauve (37). The divergent *B. cytotoxicus* strain NVH391-98 was included as an outgroup. A maximum likelihood relationship dendrogram was then built using MEGA-X version 10.0.5 (76), with 500 bootstrap replicates.

Insecticidal Cry-, Cyt-, and Vip-coding genes were annotated by both Prokka and the online pipeline BtToxin_scanner (77). The two annotations were cross-checked and protein sequences verified manually by BLAST.P and by the NCBI Conserved Domain Architecture Retrieval Tool (CDART [40]). Corresponding proteins were compared to the B. thuringiensis nomenclature database (https://www.bpprc.org/ [7]) and assigned a rank according to the following thresholds based on protein sequence identity (ID): rank 1 (i.e., Cry2), ID of >45%; rank 2 (i.e., Cry2A), ID of >78%; rank 3 (i.e., Cry2Ab), ID of >95%, and rank 4 (i.e., Cry2Ab1), ID between 95 and 100% (8). Potential bioactive compounds clusters were predicted with the online tool antiSmash v5.0 (https://antismash.secondarymetabolites.org/#!/start [78]). Mobile genetic elements (MGEs) were annotated as detailed by Fayad et al. (31).

### Whole-crystal protein identification.

In order to identify the proteins composing the H3 crystal, H3 spores and crystals were first separated using a hexane-based method as described by Rahbani Mounsef et al. (79). Hexane treatment was repeated until a 1:10 spore/crystal ratio was obtained. The purified crystal pellet was then solubilized with 50 µl of NaOH 50 mM at 30°C for 2 h and loaded on a stacking SDS-PAGE gel. After a brief migration and Coomassie brilliant blue staining, the protein band containing the entire H3 crystal was cut and analyzed by nano-LC-MS/MS (44) in the proteomic platform IGBMC in Strasbourg, France. Identified peptides were then cross-referenced with the UniProt B. thuringiensis database (http://www.uniprot.org/uniprot/?query=taxonomy:1428).

### Data availability.

Genome sequences were deposited in the NCBI database under BioProject ID PRJNA611745 and GenBank accession numbers CP052061 to CP052064.

## Supplementary Material

Supplemental file 1
