# Supplementary material for: A Novel Antidipteran Bacillus thuringiensis Strain: Unusual Cry Toxin Genes in a Highly Dynamic Plasmid Environment
Source: Appl Environ Microbiol. 2021 Feb 12;87(5):e02294-20. doi: 10.1128/AEM.02294-20 (PMC8090892; doi:10.1128/AEM.02294-20)
Supplement: Supplemental file 1 [file AEM.02294-20-s0001.pdf]

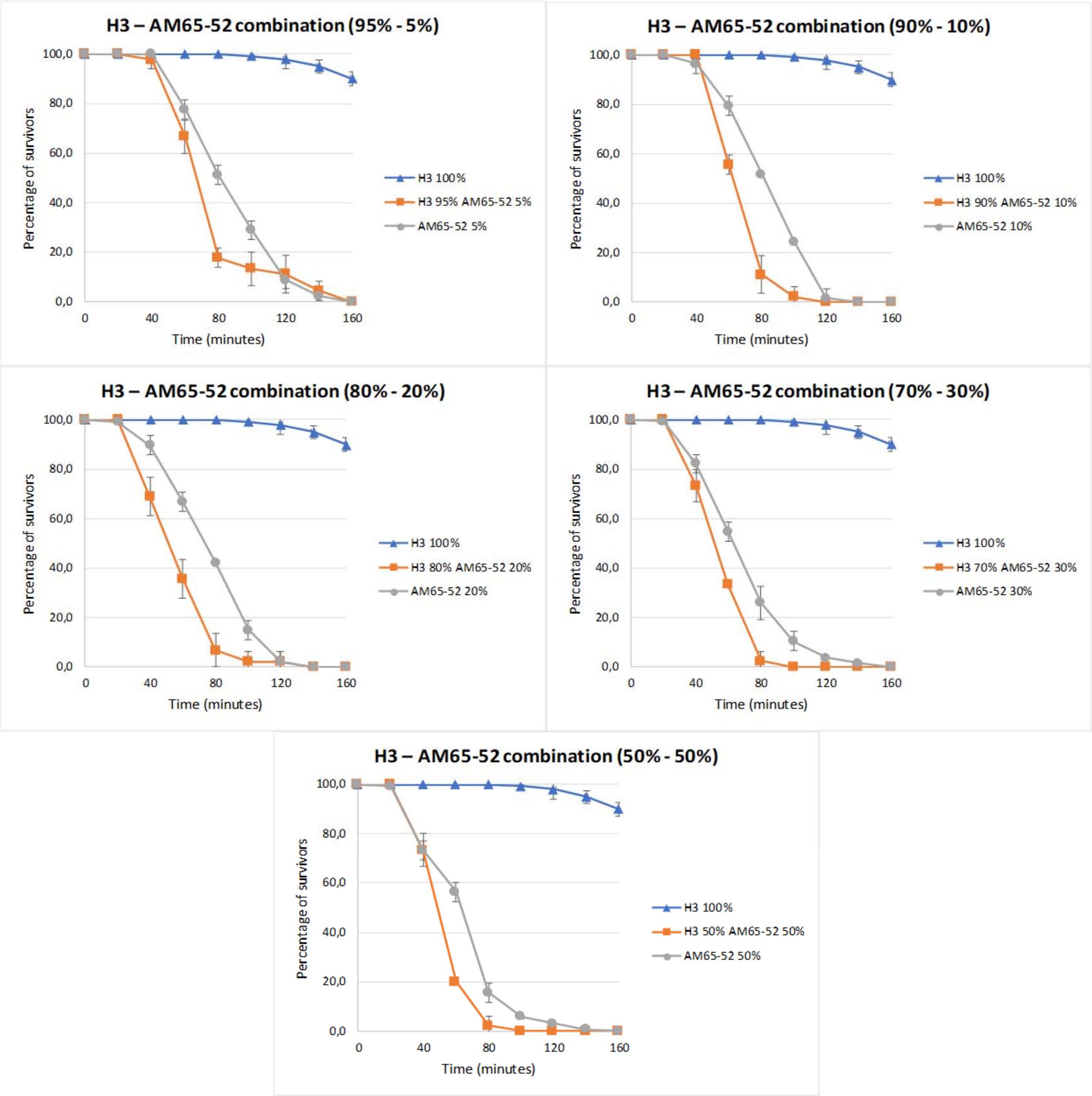

Supplementary Figure S1

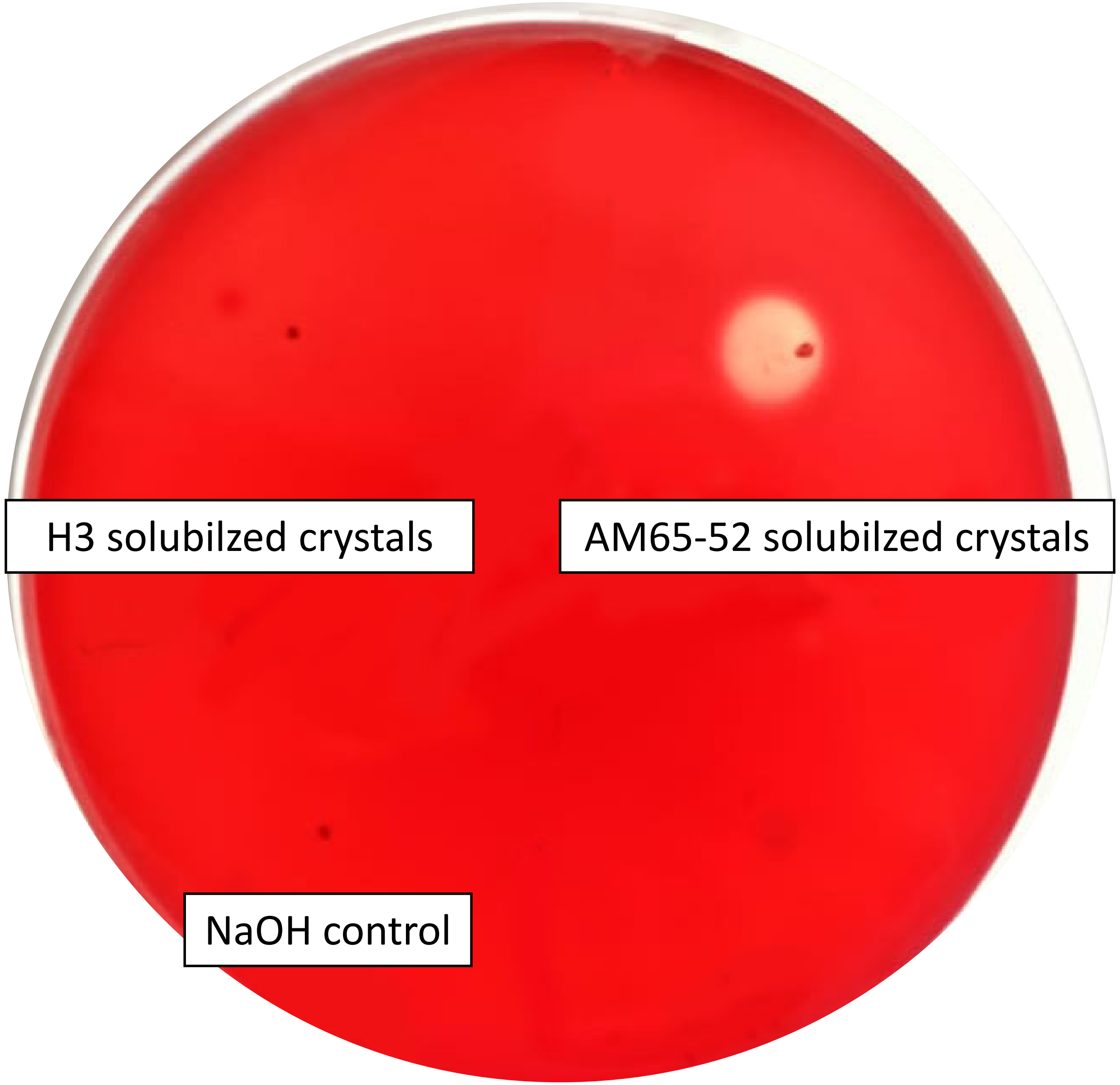

Supplementary Figure S2

```
              651     660       670       680       690       700       710       720       730       740       750       760       770       780
              |--------+---------+---------+---------+---------+---------+---------+---------+---------+---------+---------+---------+---------|
 H3-toxin-3:       MFTNYQNKVLKIETTDYEIDQAAISIECMSDEQNPQEKMMLWDEVKQAKQLSRSRNLLQNGDFGDSSGDNWTFSDDIIGSNNPIFKGNFLQIRGARDIYGTIFPTYIYQKIDESQLKPYTRYRV
 H3-toxin-7:       MYTNTMKNALKIEKTDYEIDQAAISIECMSNEQHPQEKMTLWDEVKQAKQLSRSRNLLYNGDFEDTS-NGWKTSYTIEIRENSPIFKGHYLHMFGARDIDGTLFPTYIYQKIEESKLKPYTRYRV
H3-toxin-11:       MFTSGTKNTLKIETTDYEIDQAAIAIECMSDEHNPKEKLMLWDEVKQAKQLSRSRNLLYNGDFEDSS-NGWTTSDRITIQANNPIFKGHYLNMSGARNIDGSIFPTYIYQKIDESKLKPYTRYRV
 H3-toxin-8:       MFTSGAKSMLKIETTDYEIDQAAISIECMSNEHSPKEKMILWDEVKQAKQLSWSRNLLYNGDFENTS-NGWKTSYTIEIRENSPIFKGHYLHMFGARDIDGILFPTYIYQKIEESKLKPYTRYRV
    Cry4Ba5:  EVVNALFTNDAKDALNIGTTDYDIDQAANLVECISEELYPKEKMLLLDEVKNAKQLSQSRNVLQNGDFESAT-LGWTTSDNITIQEDDPIFKGHYLHMSGARDIDGTIFPTYIFQKIDESKLKPYTRYLV
   Consensus  .....$FTn.ak..LkIeTTDY#IDQAAis!ECmS#E..PkEKM.LwDEVK#AKQLS.SRNlLqNGDFe..s..gWttSd.I.I.e#.PIFKGh%Lhm.GARDIdGtiFPTYI%QKI#ESkLKPYTRYrV

              781     790       800       810       820       830       840       850       860       870       880       890       900       910
              |--------+---------+---------+---------+---------+---------+---------+---------+---------+---------+---------+---------+---------|
 H3-toxin-3:  RGFVGSSKDLKLMVTRYGKEIDAIMDVPNDLTYMQPSP-SCGDDHRCESSSQY-VSQGYP----DEYASDMYSCPSNLGTKHVMCHDRHPFDFHIDTGELDTNTNSGIWVLFKISNPDGYATLGNLEVIE
 H3-toxin-7:  RGFVESSKDLKLVVTRYGKDIDAHMDVPNDLSYMQSRP-SCGDYNRCESPSQSGMNQGYPTPYTDGYAPDMYACPSNIDRKQVKCHDRHPFDFHIDTGELDANTNVGIDVLLKISNPDGYATLGNIEVIE
H3-toxin-11:  RGFVGSSKDLKLMVTRYGEEINGSMDVPDDLAYMQSNS-SCGDYHCCESSSQFVINQGYPTPVTDGYASDMYVCPSNLGEKHVMCHDRHPFDFHIDTGELDTNTNVGIDVLFKISNPNGYATLGNLEVIE
 H3-toxin-8:  RGFVGSSKDLKLVVIRYGKEIDAIMDVPNDLAYMQPNP-SCGDY-RCESSTQY-VSQGYPTSVTAGYAPNIYACPSNSGKKHVMCHNRHPFDFHIDTGELDTNTNIGIDVVFKISTPDGYATLGNLEVIE
    Cry4Ba5:  RGFVGSSKDVELVVSRYGEEIDAIMNVPADLNYLYPSTFDCEGSNRCETSA-------VPANI-GNTSDMLYSCQYDTGKKHVVCQDSHQFSFTIDTGALDTNENIGVWVMFKISSPDGYASLDNLEVIE
   Consensus  RGFVGSSKDlkLvV.RYGkEIDAiM#VPnDL.Y$qpsp.sCgd..RCEsS.q....qgyP......ya...YsCps#.GkKHVmCh#rHpFdFhIDTGeLDTNtNiG!wV.FKIS.PDGYAtLgNLEVIE

              911     920       930       940       950       960       970       980       990       1000      1010      1020      1030      1040
              |--------+---------+---------+---------+---------+---------+---------+---------+---------+---------+---------+---------+---------|
 H3-toxin-3:  EGPLTGEALVHVQQKEKKWNQHMEKKRSETQQAYDPAKLEVDALFTNVQGEELHYHTTLDHIQNADQLVQSIPYYVHNTWLPDVPGMNYDLYTDLHARIAQARYLYDARNVIKNGDFTQGLQGWHATGNAA
 H3-toxin-7:  EGPLTGEALAHVKHKEKKWKQHMEKKRMETQQAYDPAKQAVDTLFTDEQ--ELHYHITLDHIQNANQLVQSIPYYVHHDWLPDAPGMNYDVYQELNARIMQAYNLYDVRNVITNGDFTQGLQGWHVTGKVD
H3-toxin-11:  EGPLTGEALAHVKQKEKKWNQHMEKKRMETQQAYDPAKQAVDALFTNEQ--ELQYHITLDHIQNADQLIQSIPYYVHHEWLSDIPGVNYDLYTNLKVRIAQARYLYNARNVITNGDFTQGLLGWHVTGKVA
 H3-toxin-8:  EGPLTGEALAHVKHKEKKWNQHMEKKRMETQQAYDPAKQAVDALFTNTQGEELHYHTTLDQIKNADHLVRSIPYYVHHAWLPDVPGMNYDLYTDLSARIMQARYLYDARNVITNGDFTQGLMGWHATGKVA
    Cry4Ba5:  EGPIDGEALSRVKHMEKKWNDQMEAKRSETQQAYDVAKQAIDALFTNVQDEALQFDTTLAQIQYAEYLVQSIPYYVYNDWLSDVPGMNYDIYVELDARVAQARYLYDTRNIIKNGDFTQGVMGWHVTGNAD
   Consensus  EGPltGEAL.hVkhkEKKWN#hMEkKRsETQQAYDpAKqa!DALFTNvQ.eeLh%htTLdqIqnA#.LVqSIPYYVhn.WLpD!PGMNYDlYt#L.AR!aQARYLYDaRN!IkNGDFTQGlmGWHvTGnaa

              1041    1050      1060      1070      1080      1090      1100      1110      1120      1130      1140      1150      1160      1170
              |--------+---------+---------+---------+---------+---------+---------+---------+---------+---------+---------+---------+---------|
 H3-toxin-3:  VQQMDGASVLVLSNWSAGVSQNLNAQDHHGYVLRVIAKKEGHGKGYVTMMDCNGNQEALKFTSCEEGYMTKTVEVFPESDRVRIEIGETEGTFYIDSIELLCMKGYNSNYNQNTGTMYEQNYAHNDEQYS
 H3-toxin-7:  VQQIDGASVLVLSNWSAGVSQNLHAQDHHEYMLRVSAKKEGPGKGYVTMMDCNGKQETLKFTSCEEGYMTKTVEVFPENDRVRIEIGETEGTFYIDSIELLCMQGYASNNNSHSGNMYEQSYNGNYNQNT
H3-toxin-11:  VQQMDGASVLVLSNWSAGVSQNLNAQDHHGYVLRVIAKKEGPGKGYVTMMDCNGKQETLKFTSCEEGYVTKTVEVFPESDRVRIEIGETEGTFYIDSIELLCMQGYASNNNPHTGNMYEQ----NYNQNT
 H3-toxin-8:  VQQMDGASVLVLSNWSAGVSQNLHAQHHHGYMLRVIGKKEGPGKGYVTMMDCNGKQETLKFTSCEEGYMTKTVEVFPESDRVRIEIGETEGTFYIDSIELLCMKG
    Cry4Ba5:  VQQIDGVSVLVLSNWSAGVSQNVHLQHNHGYVLRVIAKKEGPGNGYVTLMDCEENQEKLTFTSCEEGYITKTVDVFPDTDRVRIEIGETEGSFYIESIELICMNE
   Consensus  VQQmDGaSVLVLSNWSAGVSQNlhaQhhHGYvLRVIaKKEGpGkGYVT$MDC#gnQE.LkFTSCEEGYmTKTV#VFP#sDRVRIEIGETEGtFYI#SIELlCM.g.........................

              1171    1180      1190      1200      1210      122022
              |--------+---------+---------+---------+---------+-|
 H3-toxin-3:  GCTCNQGHNNNYNQDS----------------GCKCNQGYNS
 H3-toxin-7:  SDVYHQGYTNNYNQDSSSMYNQNYTNNDDLHSGCTCNQGHNSGCTCNQGYNR
H3-toxin-11:  SDVYHQGYTNNYNQDSSRVLLQSFLSEVKM
 H3-toxin-8:
    Cry4Ba5:
   Consensus  ..............................  ..........
```

Supplementary Figure S3

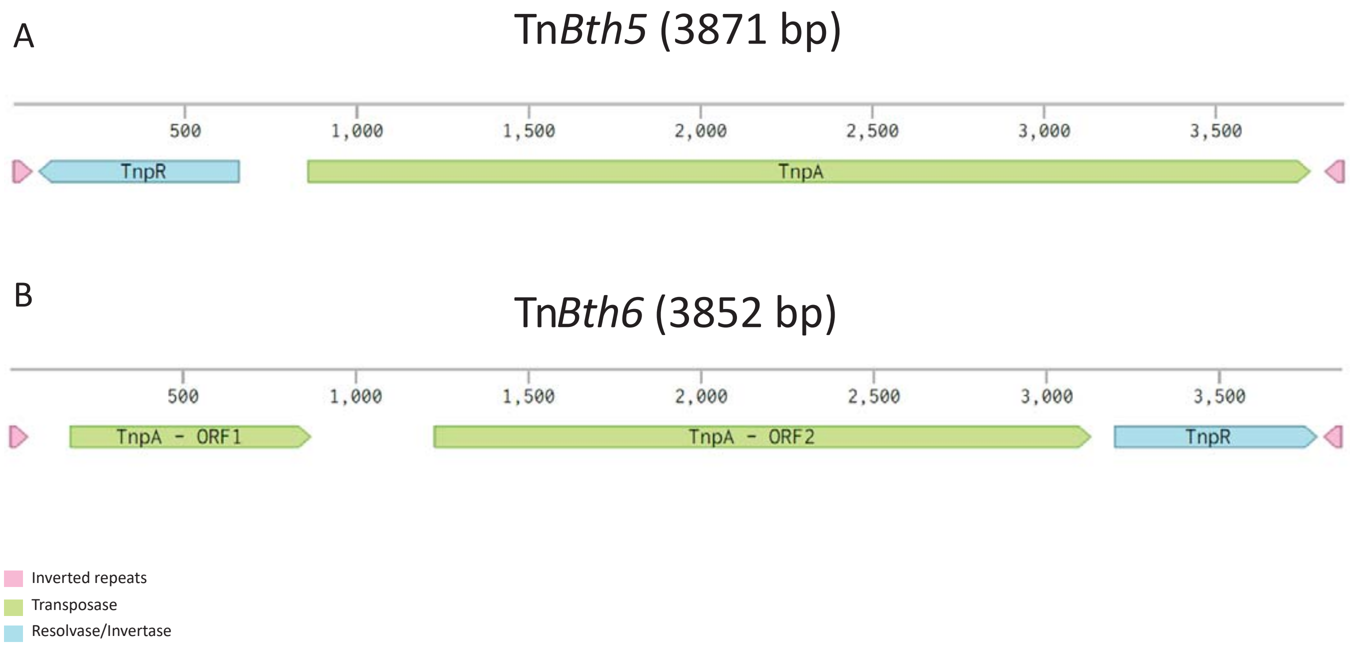

Supplementary Figure S4

Fig. S1 Comparative activity tests between AM65-52 and H3 crystal-spore mixtures (40 µg/mL) with different ratios on third instar larvae of *A. albopictus*. The graphs show the percentage of survivors through time for the combination H3–AM65-52, AM65-52 alone and H3 alone. These results are representative of biological and experimental triplicates.

Fig. S2 Testing of 10 µL of washed, solubilized and filtered H3 and AM65-52 crystals on 5% sheep blood agar plates. Plates were incubated at room temperature and observed after 4 hours.

Fig. S3 Multiple sequence alignments of the four proteins annotated as ORF2 with the C-terminal part of Cry4Ba5, produced by the reference *B. thuringiensis* sv. *israelensis* strain AM65-52. Red and blue indicate more or less conserved amino acid residues. Alignment was done using Multialin (http://multalin.toulouse.inra.fr/multalin/).

Fig. S4 Annotated schema of the two Tn*3* family class II transposable elements, showing terminal inverted repeats, flanking transposase and resolvase coding genes. Annotations were made with Benchling editor (https://benchling.com/editor).

Table S1. IS, Tn and intron-like elements found on the three plasmids of strain H3.

Element	Complete (Yes/No)	Type/ Family	Orientation	Start*	End*	Length (bp)	Number of CDSs	IR (bp)	DR (bp)	Remarks
**pH3-552**										
IS*Bce3*-like	Yes	IS*200*/IS*605*	-	35,113	36,754	1,642	2	NA	0	The two CDSs correspond to a Transposase and an accessory protein
IS*Bth167*-like	Yes	IS*3*	-	91,480	92,916	1,474	2	24/25	3	The two CDSs form a fusion protein following -1 frameshifting
IS*Bth167*-like	Yes	IS*3*	-	130,037	131,473	1,474	2	24/25	3	The two CDSs form a fusion protein following -1 frameshifting
IS*Bce14*-like	Yes	IS*3*	+	264,027	265,484	1,458	2	16/22	0	The two CDSs form a fusion protein following -1 frameshifting
IS*Bce14*-like	Yes	IS*3*	+	370,770	372,213	1,444	2	22/30	0	The two CDSs form a fusion protein following -1 frameshifting
IS*231A*-like	Yes	IS*4*	+	383,901	385,553	1,653	1	20/20	12	-
IS*231A*-like	Yes	IS*4*	+	427,182	428,834	1,653	1	20/20	11	-
IS*Bth17*-like	Yes	IS*200*/IS*605*	+	490,170	491,514	1,345	2	NA	0	The two CDSs correspond to a Transposase and an accessory protein
**pH3-180**										
IS*240A*-like	Yes	IS*6*	-	1,831	2,690	860	1	16/17	0	-
IS*Bth20*-like	Yes	IS*6*	+	3,905	4,712	808	1	23/27	2	-
IS*Bth20*-like	Yes	IS*6*	-	12,542	13,350	809	1	23/27	0	-
IS*240A*-like	Yes	IS*6*	+	13,450	14,258	860	1	16/17	0	-
IS*240A*-like	Yes	IS*6*	+	18,057	18,521	793	1	15/16	2	-
IS*Bth166*-like	Yes	IS*110*	+	26,499	27,728	1,229	1	x	2	No IR are present for the reference element in the ISFinder database (Accession number: DQ242517)
IS*Bth20*-like	Yes	IS*6*	-	29,096	29,904	809	1	22/24	3	-

IS*Bth20*-like	Yes	IS*6*	-	35,861	36,631	809	1	22/25	0	-
IS*Bs2*-like	Yes	IS*630*	+	37,789	39,017	1,102	2	11/12	2	The two CDSs form a fusion protein following -1 frameshifting
IS*Bth20*-like	Yes	IS*6*	-	47,312	48,132	812	1	23/27	0	-
IS-H3-new.1	No	PD-(D/E)XK	-	48,668	49,561	893	1	x	NA	New putative family with no known IR
IS*Bth20*-like	Yes	IS*6*	-	59,734	60,542	809	1	23/27	3	-
IS*Bth20*-like	Yes	IS*6*	+	63,614	64,422	809	1	22/25	2	-
IS*232*-like	Yes	IS*21*	+	74,271	76,653	2,383	2	15/25	0	One CDS corresponds to the transposase, the second encodes a helper protein
IS-H3-new.2	Yes	IS*L3*	+	76,665	77,707	1,042	2	x	0	-
IS*240A*-like	Yes	IS*6*	+	79,775	80,634	860	1	16/17	2	-
IS*Bth20*-like	Yes	IS*6*	+	85,617	86,424	808	1	21/22	0	-
IS*Cce4*-like	Yes	IS*3*	+	90,177	91,436	1,260	2	19/23	0	The two CDSs form a fusion protein following -1 frameshifting
IS*Bth167*-like	Yes	IS*3*	-	94,227	95,663	1,483	2	16/16	3	The two CDSs form a fusion protein following -1 frameshifting
IS*231A*-like	Yes	IS*4*	+	101,217	102,869	1,653	1	20/20	0	
IS*Cce4*-like	Yes	IS*3*	+	107,398	108,654	1,260	2	19/23	0	The two CDSs form a fusion protein following -1 frameshifting
IS*Cce4*-like	Yes	IS*3*	-	121,012	122,268	1,260	2	19/23	0	The two CDSs form a fusion protein following -1

										frameshifting
IS*232*-like	Yes	IS*21*	+	123,454	125,836	2,383	2	15/25	0	One CDS corresponds to the transposase, the second encodes a helper protein
IS*240A*-like	Yes	IS*6*	-	135,843	136,702	860	1	15/17	2	-
IS*Bth20*-like	Yes	IS*6*	+	137,580	138,388	809	1	23/27	2	-
IS*Bth20*-like	Yes	IS*6*	+	143,251	144,059	809	1	23/27	2	-
IS*Bs2*-like	Yes	IS*630*	+	144,102	145,240	1,139	2	`9/12	0	The two CDSs form a fusion protein following -1 frameshifting
IS*Bth20*-like	Yes	IS*6*	+	157,853	158,661	809	1	23/27	2	-
IS*Bth20*-like	Yes	IS*6*	-	163,707	164,515	809	1	23/27	0	-
IS*Bs2*-like	Yes	IS*630*	+	167,267	168,405	1,139	2	`9/12	2	The two CDSs form a fusion protein following -1 frameshifting
IS*Bth165*-like	Yes	ISNCY	-	168,607	169,615	1,009	1	13/21	0	-
IS*Bth8*-like	Yes	IS*3*	+	179,382	180,744	1,362	2	17/23	2	The two CDSs form a fusion protein following -1 frameshifting
Tn*Bth5*	Yes	Tn*3*	-<br>+	14,194	18,064	3,871	2	49/50	5	The two CDSs correspond to a 1,005 aa transposase and a 193 aa resolvase
Tn*Bth6*	Yes	Tn*3*	+	169,513	173,364	3,852	3	48/50	5	Two of the three CDSs correspond to a disrupted 922 aa transposase and a 194 aa resolvase/invertase
Tn*4430*-like	No	Tn*3*	-	4,691	7,420	2,703	1	x	NA	Only a transposase gene without the associated resolvase. No IR were found in proximity

*B.th.*I3-like	No	Group II Intron	-	11,437	12,441	1,004	1	NA	NA	The ORF corresponds to the Intron encoded protein IEP
**pH3-101**										
IS-H3-new.3	No	Rpn family putative transposase	+	1,011	2,809	1,798	1	x	NA	New putative family with no known IR
IS*Bce14*-like	Yes	IS*3*	+	16,563	28,020	1,458	1	8/10	0	
Tn*Bth5*	Yes	Tn*3*	- / +	72,867	76,737	3,871	2	49/50	5	The two CDSs correspond to a 1,005 aa transposase and a 193 aa resolvase

* Start and End refer to the coordinates of the elements, irrespective of the transposase orientation. In case of undetected IR, Start and End indicate the positions of the ORF(s).

x = no IR were found in proximity of the transposase gene.

NA = Not Applicable.

Table S2. Potential composite transposons, with sizes less than 10 kb, formed by IS*Bth20*-like elements on pH3-180.

Potential composite transposon	Passenger genes in order (excluding hypothetical proteins)*	Isoforms**	Start[#]	End[#]	Length (bp)
Tn*H3.1*	Tn*4430*-like transposase<br>*cry70B*-like<br>*B.th.*I3-like	Yes	3,905	13,350	9,445
Tn*H3.2*	*cry54*-like<br>*orf2*<br>*phosphatidylinositol phosphodiesterase precursor*	Yes	29,096	36,631	7,535
Tn*H3.3*	*phosphatidylinositol phosphodiesterase precursor*	No	59,734	64,422	4,688
Tn*H3.4*	*Glucose-6-dehydrogenase*<br>*Glucose-1-phosphatase*	Yes	137,580	144,059	6,479
Tn*H3.5*	*dTDP-glucose-2C6-dehydratase*<br>*GDP-mannose-dependent alpha-(1-6)-phosphatidylinositol monomannoside mannosyltransferase*	Yes	157,853	164,515	6,662

* Ascending order in positions on the plasmid.

** Isoforms indicate that the two elements show more than 95% DNA or 98% amino acid identity.

[#] Positions correspond to the start/end of the outer ISBth20 elements.

10

2

Table S3. Identified peptides following nano-LC MS/MS analysis of solubilized pure H3 crystal, and their position in each crystal protein.

Peptide * / H3 toxins	Cry70B-like	Cry54-like	ORF2	Cry68-like	3D Cry H3-1#	Cry71-like	ORF2	ORF2	3D Cry H3-2	Cry50-like	ORF2
HPFDFHIDTGELDTNTNVGIDVLFK			208-232				212-136	210-234			212-236
HVmcHDRHPFDFHIDTGELDTNTNVGIDVLFK			201-232				205-236	203-234			205-236
DIYGTIFPTYIYQK			98-111				97-110	97-110			97-110
IMQAYNLYDVR							349-359	349-359			
NLLQNGDFGDFSGNDWTFGNDIIIGSNNPIFK			56-87								
INEITQVAAVNTR				515-527							
NVNISAPTDVSGVISNR				380-396							
ITFGNGSSQVIPLVSTTSSLNDLQYESFR				108-118							
IALDVTFSLANR				167-178							
FLPGENSDTPTPQDYTHLLSR				240-247							
VVNATVGLTPATGNQR				458-478							
FLPGENSDTPTPQDYTHLLSRVVNATVGLTPAT GNQR				458-494							
LIDQALENQIR				479-494						8-24	
NEYEILNASPNHEYMSNR				506-514							
VISGPGFTGGDLVR		554-566		534-547<br>203-209	542-555	531-543			541-533	523-535	
mYSNYcVR				586-614<br>628-642	594-622						
SLTSENIYR											
NVITNGDFTQGLMGWHVTGK			358-376				360-379<br>56-73	360-379<br>56-73			360-379<br>56-73
EIDAIMDVPNDLAYMQPSPScGDAHR			145-170				144-169	144-166			
NLLYNGDFEDTSNGWK			56-67<br>358-372				56-71<br>360-374	56-71<br>360-374			56-70<br>360-374

GPGHTGGDLVALTSNGTLSGR	788-793<br>420-431						544-555<br>86-104	526-546
VIKGPGHTGGDLVALTSNGTLSGR							541-555<br>83-104	523-546
DVHLFTWLK								338-346
TYQEGLNILK		284-293					272-281	
YFDIWNNNK		159-167						
IDYDKITQIPAVK					510-522		519-531	
IEFIPITQSVLDYTEEQNLETAQEVVDNLFIN								657-688
VSFAWTHR		517-523						486-493
ISNPDGYATLGNLEVIEEGPLTGEALAHVK		233-262			237-266	235-264		237-266
LETTDYEIDQAAISIEcMSDEQDLQEK		12-38			12-38	12-38		12-38<br>299-305
IDESKLKPYTR		112-122			111-121	111-12		111-121
METQEAYDPAK		278-287			281-291	29-289		281-291
TmLWDEVK		40-46				40-46		40-46
IEIGETEGSFYIESIELNcmK		463-483			465-485	465-485		465-485
FTScEEGYmTK		440-450<br>529-533			422-454<br>556-561	442-454		442-452
MILWDEVK	796-802							
MmLWDEVK		39-46			39-46	39-46		39-46
GYVTMmDcNGNQETLK		424-439			426-441	426-441		426-441
ITQIPAVK		537-544		526-533	515-522		524-531	506-513
LRGTNASSWIR					253-263			
TVEVFPESDRVR		451-460			453-462	453-462		453-462

* Color intensity indicates the increasing numbers of time the corresponding peptide was found according to the final nano-LC MS/MS analysis.
